# Endothelial cells loss to the hyperopic pacients during phacoemulsification

**Published:** 2017

**Authors:** Dan-Mircea Stănilă, Andreea-Maria Florea, Adriana Stănilă, Alina Adriana Panga

**Affiliations:** * Department of Ophthalmology, Clinical Emergency Hospital Sibiu, Sibiu, Romania; ** Faculty of Medicine, Lucian Blaga University, Sibiu, Romania; *** Ocular Surface Research Center, Sibiu, Romania; **** Ofta Total Clinic Sibiu, Romania

**Keywords:** hyperopia, shallow anterior chamber, phacoemulsification, endothelial cells, specular microscopy

## Abstract

****Introduction**:**

The phacoemulsification cataract surgery is the most frequently performed surgery and it generally improves vision in over 90% of the patients. Hyperopic patients are a challenge during phacoemulsification especially because of their short eyeball and shallow anterior chamber. A shallow anterior chamber is associated with overall reduction of the safe zone, which may lead to difficulty in creating the corneal incisions, harder capsulorhexis performing, or endothelial complications.

****Purpose**:**

The aim of the study was to present the endothelial cells loss after the phacoemulsification procedure in the hyperopic patients.

****Material and Methods**:**

A number of 1775 patients operated in the Ophthalmology Department of the Clinical Hospital Sibiu from January 11, 2011 to December 20, 2013 have been included in our study; 595 cases with emmetropia and the rest of the 1180 patients had the following refraction errors: 216 - myopia and 964 - hypermetropia. From the total cases of the hypermetropia, we selected 72 patients to measure the endothelial cells density and the corneal thickness by using specular microscopy, one day before and 7-14 days after surgery.

****Results and discussions**:**

The preexisting hypermetropia might modify the intraoperative and postoperative cataract surgery evolution. Endothelial cell loss is potentially higher from surgical trauma so that the endothelium must be protected with viscoelastics. The loss of endothelial cells in hyperopic eyes occurred with an average of 267 cell/ mm² and the thickness of the cornea increased by 13 µm.

****Conclusion**:**

The phacoemulsification surgery in the presence of hypermetropia requires more attention. The biometry and the specular microscopy are very important tasks for the preoperative assessment, surgery, and postoperative care. The protection of the corneal endothelium with viscoelastics leads to an insignificant modification of the endothelial cells in hyperopic patients compared to an anterior study of the patients with all ametropies.

## Introduction

The phacoemulsification cataract surgery is the most frequently performed surgery in ophthalmology and it generally improves vision in over 90% of the patients.

Hyperopic patients are a challenge during phacoemulsification especially because of their short eyeball and shallow anterior chamber [**1**].

A shallow anterior chamber is associated with the overall reduction of the safe zone, which may lead to difficulty in creating the corneal incisions, harder capsulorhexis performing or corneal complications, especially endothelial cells [**2**-**4**].

Studies showed that in 83% of the cases, the anterior chamber in hyperopic eyes is normal and in 17%, they have shallow anterior chamber [**5**].

In these cases of shallow anterior chamber, the crystalline is normal or even bigger, which leads to the movement of iridocrystalline diaphragm with the narrowing of the anterior chamber and high risk of intraocular hypertension [**2**].

The episodes of the raised intraocular pressure affect the corneal endothelium [**1**].

## Aim of the Study

The aim of the study was to present our experience at the endothelial cells (EC) loss after the phacoemulsification procedure in the hyperopic patients.

## Material and Method

**Fig. 1:**
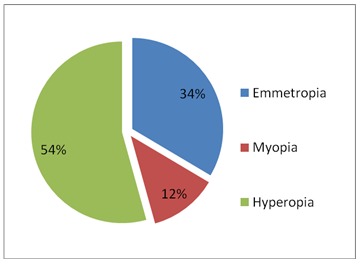
Distribution of refraction

A number of 1775 patients operated in the Ophthalmology Department of the Clinical Hospital Sibiu from January 11, 2011 to December 20, 2013 have been included in our study; 595 cases with emmetropia and the rest of the 1180 patients had the following refraction errors: 216 - myopia and 964 - hypermetropia (**Fig. 1**).

The same surgeon performed the phacoemulsification surgery with the same device. Cases of pathologic cataract have been excluded from our study. We considered the refraction errors depending on the biometry: biometry smaller than +19,5 D, myopic eyes, biometry between +20-+21,5 D emmetropic eyes and biometry bigger than +22 D, hyperopic eyes [**6**-**8**] (**Table 1**).

**Table 1:** Hyperopia degrees

Dioptric power	Patients
+22.00D - +24.00D	742
+24.50D - +26.50D	179
>+27.00 D	43

**Fig. 2:**
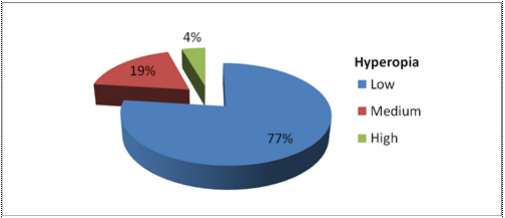
Classification of hyperopia

From the total cases of the low, medium and high hyperopia (**Fig. 2**) we selected 72 patients to measure, using specular microscopy, CSO device, the endothelial cells density and the corneal thickness, one day before and 7-14 days after surgery. We examined the loss of endothelial cells and thickness of the cornea and compared the results with an anterior study on 80 patients, which included all ametropies and emmetropia. 

## Results and Discussions

**Fig. 3:**
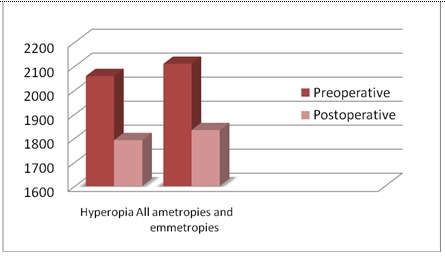
The loss of endothelial cells compared with an anterior study

The loss of endothelial cells in hyperopic eyes occurred with an average of 267 cell/ mm² compared to the anterior study of all ametropies and emmetropia, in which the average was 275 cell/ mm² (**Fig. 3**).

**Fig. 4:**
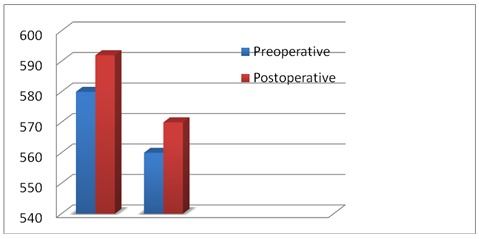
Increased thickness of the cornea compared with an anterior study

The thickness of the cornea in hyperopic eyes, increased by 13 µm, compared to the anterior study of all emmetropia and ametropies in which the thickness of the cornea increased by 10 microns (**Fig. 4**). 

The loss of endothelial cells was with 8 cells less and the thickness of the cornea raised with 3 microns.

The preexisting hypermetropia may modify the intraoperative and postoperative cataract surgery evolution [**1**].

Endothelial cell loss is potentially higher from surgical trauma so that the endothelium must be protected with viscoelastics, cohesive and dispersive hyaluronic acid substances [**2**,**9**,**10**].

A highly hypermetropic eye does not have the maximum mydriatic pupil [**2**]. 

Incisions must be properly made, with smaller length of the tunnel, and extended a bit more anteriorly to discourage iris prolapse or corneal deterioration [**1**,**11**]. 

Capsulorhexis becomes more difficult to perform because of increased vitreous pressure that causes a tendency to rip outwards, which increases the risk of posterior capsule rupture and subluxation of the lens [**1**,**11**]. 

So, the staining of the anterior capsule and the use of viscoelastics are helpful in order to balance the positive pressure of the vitreous [**1**,**11**]. 

In our study, 14 cases with shallow anterior chamber capsulorhexis were harder to perform, cumulative dissipated energy was higher and immediately in postoperative period, patients complicated with corneal edema [**3**,**4**,**12**].

Phacoemulsification: the technique stop & chop is performed for the minimization of the zonular stress and the deterioration of the corneal endothelium. Also, high energies of ultrasound must be avoided [**1**]. 

In the category of hyperopic patients, with or without astigmatism, we took into consideration the patient’s option for postoperative refraction.

The hyperopic patients wished improvement in both near and distant vision.

Postoperative, all patients preferred to improve their distant vision without optical correction, continuing to wear glasses for reading. 

Some patients had chosen multifocal IOLs for both nearsightedness and farsightedness.

**Fig. 5:**
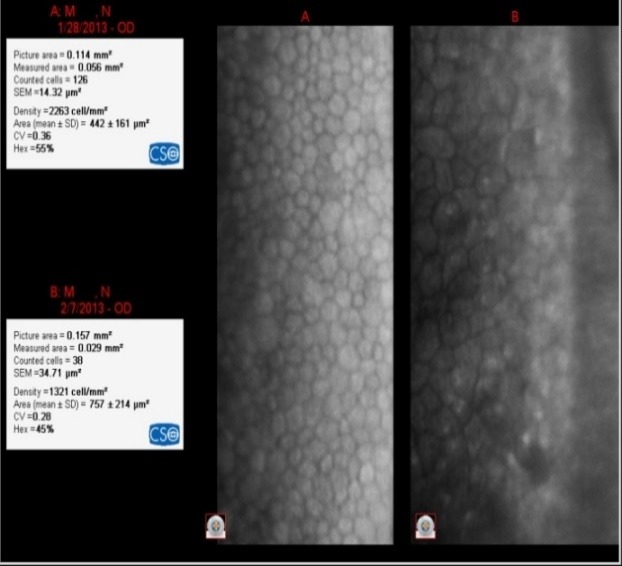
IOL= +22D The loss of EC was 942 cell/ mm²

**Fig. 6:**
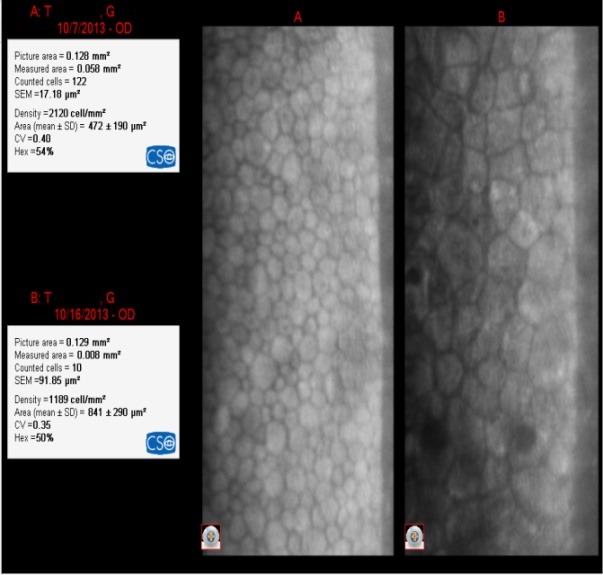
IOL= +22.50D The loss of EC was 931 cell/ mm²

**Fig. 7:**
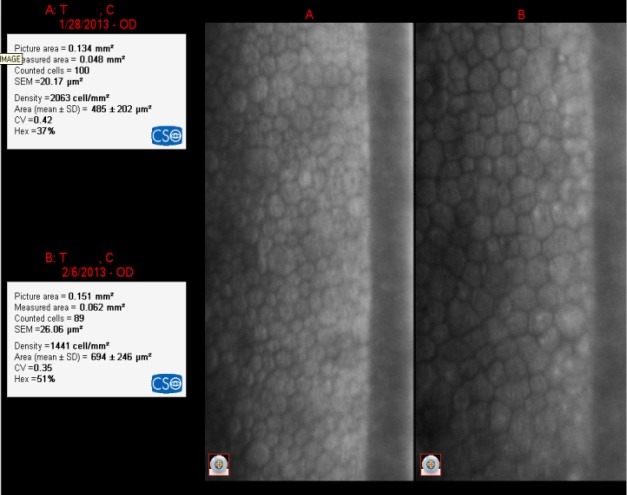
IOL= +26.50D The loss of EC was 622 cell/ mm²

**Fig. 8:**
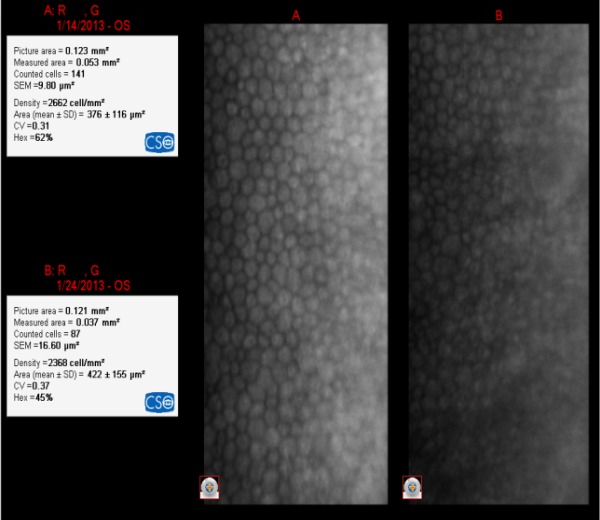
IOL= +28.50D The loss of EC was 294 cell/ mm²

**Fig. 9:**
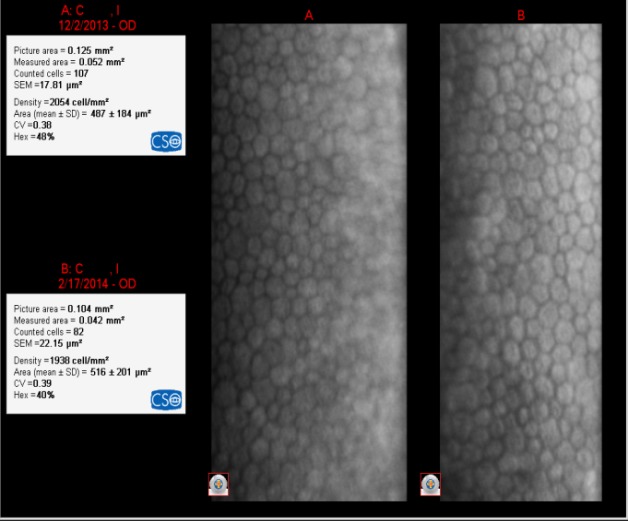
IOL= +29.50D The loss of EC was 116 cell/ mm²

In most of the cases, women were the ones who opted for an implanted multifocal IOL in order to abandon glasses completely. In **Fig. 5**-**9**, we showed the endothelial cells before and after surgery. It was not a correlation between the degree of hypermetropia and the endothelial cells loss after surgery.

**The loss of endothelial cells related to the degree of hyperopia and the power of the IOL** (**Fig. 5**-**9**)

## Conclusion

The phacoemulsification surgery in the presence of hypermetropia requires more attention.

The biometry and the specular microscopy are very important tasks.

The protection of the corneal endothelium with viscoelastics is necessary.

There was an insignificant loss of the cells in hyperopic patients compared to an anterior study of the patients with all ametropies and emmetropia. 

The loss of endothelial cells was with 8 cells more and the thickness of the cornea raised with 3 microns after surgery.

It was very important to take into consideration the option of the patients related with postoperative refraction.
